# CIP2A Influences Survival in Colon Cancer and Is Critical for Maintaining Myc Expression

**DOI:** 10.1371/journal.pone.0075292

**Published:** 2013-10-01

**Authors:** Armin Wiegering, Christina Pfann, Friedrich Wilhelm Uthe, Christoph Otto, Lukas Rycak, Uwe Mäder, Martin Gasser, Anna-Maria Waaga-Gasser, Martin Eilers, Christoph-Thomas Germer

**Affiliations:** 1 Department of General, Visceral, Vascular and Paediatric Surgery (Department of Surgery I), University Hospital Wuerzburg, Wuerzburg, Germany; 2 Department of Biochemistry and Molecular Biology, University of Wuerzburg, Wuerzburg, Germany; 3 Institute of Molecular Biology and Tumor Research, University of Marburg, Marburg, Germany; 4 Comprehensive Cancer Center Mainfranken, University of Wuerzburg, Wuerzburg, Germany; University of Illinois, United States of America

## Abstract

The cancerous inhibitor of protein phosphatase 2A (CIP2A) is an oncogenic factor that stabilises the c-Myc protein. CIP2A is overexpressed in several tumours, and expression levels are an independent marker for long-term outcome. To determine whether *CIP2A* expression is elevated in colon cancer and whether it might serve as a prognostic marker for survival, we analysed *CIP2A* mRNA expression by real-time PCR in 104 colon cancer samples. *CIP2A* mRNA was overexpressed in colon cancer samples and *CIP2A* expression levels correlated significantly with tumour stage. We found that *CIP2A* serves as an independent prognostic marker for disease-free and overall survival. Further, we investigated CIP2A-dependent effects on levels of c-Myc, Akt and on cell proliferation in three colon cancer cell lines by silencing CIP2A using small interfering (si) and short hairpin (sh) RNAs. Depletion of CIP2A substantially inhibited growth of colon cell lines and reduced c-Myc levels without affecting expression or function of the upstream regulatory kinase, Akt. Expression of CIP2A was found to be dependent on MAPK activity, linking elevated c-Myc expression to deregulated signal transduction in colon cancer.

## Introduction

Colorectal cancer (CRC) is the most common gastrointestinal malignancy. There are approximately 664,000 new cases each year worldwide. Half of these patients will die from this carcinoma [1]. Currently, standard treatment is primary surgery and, depending on the tumour stage, additional chemotherapy [2]. Due to the high recurrence rate, new potential targets and prognostic markers are needed to identify patients that are likely to benefit from additional therapy.

In 2007, Junttila and Westermarck identified the cancerous inhibitor of protein phosphatase 2A (CIP2A) as a human oncoprotein. CIP2A is overexpressed in head and neck squamous cell carcinomas and in colon carcinomas [3]. CIP2A inhibits the protein phosphatase 2A (PP2A). PP2A in turn has a critical role in turnover of the c-Myc oncoprotein, since PP2A dephosphorylates c-Myc at serine-62 (S62). Dephosphorylation at S62 is required for ubiquitination of c-Myc by the ubiquitin ligase Fbw7 and therefore, initiates degradation of c-Myc [4]. Overexpression of CIP2A inhibits PP2A activity and thereby stabilizes c-Myc. Consequently, this induces immortalisation and malignant transformation of human cells [3]. c-Myc itself remains the most important oncogenic driver in colorectal cancer [5].

Recent studies have shown that CIP2A is overexpressed in several human malignancies. CIP2A expression levels correlate with overall survival (OS) and disease free survival (DFS) in gastric carcinomas, in serous ovarian cancer, in renal cell carcinoma and in breast cancer [6]; [7]; [8]; [9]. In chronic myeloid leukemia (CML), *CIP2A* expression at the time point of diagnosis is a prognostic marker for the development of a blast crisis later on [10]. Furthermore, some oncogenic factors, including *helicobacter pylori* and papilloma virus 16 E7, upregulate expression of CIP2A and this may be critical for their oncogenic activity [11]; [12].

Recently, two groups reported conflicting results regarding the prognostic impact of CIP2A in colorectal cancer [13], [14]. Böckelman et al. studied 752 patients, but could not determine any prognostic significance; in contrast Teng et al. studied 167 patients and identified CIP2A expression as a prognostic factor for colon carcinoma.

The aim of the present study was to address the relevance of CIP2A expression in colon cancer. First, we analysed expression of *CIP2A* in a cohort of 104 colon cancer patients with documented follow-up and confirmed its overexpression. Secondly, we investigated the association between *CIP2A* mRNA expression and clinical-pathological variables; lastly, we aimed to determine the molecular pathways that regulate CIP2A expression and, are regulated by CIP2A. Our data support the notion that deregulated expression of CIP2A is a critical oncogenic event in colon carcinoma.

## Patients and Methods

### Patient Samples

One hundred and four patients with colorectal cancer were included in the study. All patients underwent surgery at the University Hospital of Wuerzburg, Germany, between 2003 and 2012. None of the patients had received chemotherapy or radiotherapy before surgery. Treatments after surgery were performed according to guidelines for treating colorectal cancer. The diagnosis was confirmed by histopathological examination of the specimens. After surgery, tumour specimens were collected and stored in liquid nitrogen. Clinical data of all the patients were collected from hospital records and subsequent records were collected via the Comprehensive Cancer Centre Mainfranken.

### Ethics Statement

Ethical approval for this research was obtained from the Human Research Ethics Committee of the University of Wuerzburg. All patients that provided tumor tissue and normal colon tissue samples for this research signed a consent form prior to surgical removal of the intestinal cancer.

### Cell Lines and Cell Culture

Caco2, HCT116, and SW620 cells were purchased from American Type Culture Collection. All cells were cultured in DMEM supplemented with 10% fetal calf serum und 1% penicillin/streptomycin.

### Real-time Quantitative Reverse Transcription-PCR Analysis

Gene expression of *CIP2A* was analysed using real-time PCR. Total cellular RNA was extracted from tumour samples and cell lines with RNeasy Minikit (Qiagen; Hilden, Germany) according to the manufacturer’s instructions. Primer sets (Qiagen) that targeted *CIP2A* RNA were designed by Biomers (Ulm, Germany). Matched human colon cDNA (Pharmingen; Heidelberg, Germany) served as a positive control, standardized to baseline. The housekeeping genes, glyceraldehyde-3-phosphate dehydrogenase (*GAPDH*) and beta-2 microglobulin (*b2MG*) were used as internal standards for relative quantification and cDNA quality control. All PCR reactions were carried out with a DNA Engine Opticon 2 System (MJ Research, Biozym; Oldendorf, Germany). Relative quantification, based on the fold difference, was calculated with the threshold cycle (Ct) method, expressed as 2^−ΔΔCt^ (Primer sequence in Table S1).

### Immunoblot Analysis

Cultured cells were rinsed three times with ice-cold PBS, harvested, and lysed directly in RIPA sample buffer for Immunoblot analysis. Cell debris was removed by centrifugation at 12,000 g for 10 min at 4°C, and the supernatant was used as total protein lysate. For each sample, 10 µg of total protein lysate was subjected to 10% sodium dodecyl sulphate-polyacrylamide gel electrophoresis (SDS-Page), followed by Immunoblot analysis. Immunoblots were probed with antibodies against CIP2A (A301-454A; Bethyl Laboratories), c-Myc C33 (C33, #42; Santa Cruz), beta-actin (AC-15/A5441; Sigma), vinculin (V9131; Sigma), AKT, pAKT^473^ (cs-9271), gsk3 (cs-9315), pGSK Serine-9 (cs-9336), s6 (cs-2212), ps6 (cs-2215); pmTOR (cs-2917), and mTOR (cs-2972). All antibodies were from Cell Signalling and were used according to the manufacturer’s instructions. The blots were visualised with secondary antibodies (GE Healthcare) against mouse (NA9310) or rabbit (NA9340) primary antibodies.

### Immunohistochemistry

The CIP2A antibody was the same as for Immunoblot analysis, isotype control antibody was purchased from eBioscience (San Diego, USA). The secondary antibody was a Cy3-conjugated AffiniPure anti-rabbit IgG at a 1∶200 dilution. CIP2A staining was performed on cryostat sections of snap-frozen colon cancer specimens expressing different CIP2A mRNA levels with neighbouring normal colon tissue and 10 normal colon specimens. Cryostat sections (5 µm) were incubated with the primary antibody or control antibody followed by incubation with the secondary Cy3-conjugated antibody. Slides were counterstained with DAPI (4′,6-Diamidino-2-phenylindoldihydrochlorid) (Sigma-Aldrich, Steinheim, Germany) and covered with polyvinyl-alcohol mounting medium (DABCO, Sigma-Aldrich) and analysed using a Zeiss camera (Oberkochen, Germany).

### siRNA Transfection

To silence gene expression, cells were transfected with small interfering RNAs (siRNAs). On-target plus SMART pool (Dharmacon) siRNA to target CIP2A (L-014135-01-005) and a control siRNA (D-001810-10-05) were used. The siRNA pools (final concentration 100 nM) were transfected into the cells with the RNAimax kit according to the manufacturer’s protocol. Cells were harvested 72 h later; expression of proteins was determined by Immunoblot analysis.

### shRNA and Lentivirus

To silence CIP2A mRNA expression with short hairpin RNA (shRNA) sequences, targeting *CIP2A* were cloned into a lentivirus vector (plko1 puro), according to the manufacturer’s protocol. The vector was transiently transfected into HEK293t cells together with package plasmids. After 48 and 72 h, supernatants containing the virus were collected and filtered. Colon cancer cell lines were infected with the CIP2A lentivirus and 24 h later, infected cells were selected with puromycin. Experiments were performed with the selected cells.

### Colony Formation Assay

2.5×10^3^ cells infected with shRNA CIP2A or Scr. were plated on six well plates. Colonies were stained with 0.5% crystal violet in 20% ethanol. Photos were taken and relative density determined with ImageJ.

### Statistical Analysis

Univariate analyses to determine association with survival were evaluated with Kaplan–Meier curves, and comparisons were performed by Mann-Whitney U test. Multivariate analyses were performed with a Cox proportional hazards regression model. P-values <0.05 were considered statistically significant.

## Results

### Expression of CIP2A and Clinicopathological Variables of Colon Cancer Patients

Expression of *CIP2A* mRNA was initially assessed using expression data sets derived from a published microarray analysis, which was originally performed to identify prognostic markers for colorectal carcinomas according to the lymph node metastasis status [15]. Complete data sets (DFS, Tumor stages), comparing *CIP2A* mRNA expression in carcinoma to that in matched adjacent tissues, were available for 226 carcinomas. *CIP2A* expression was tested using two independent probes present on this array. We found no correlation between *CIP2A* expression and age at diagnosis, or gender in this data set, but *CIP2A* mRNA was expressed at higher levels in colorectal cancer tissues than in the matched adjacent tissues in both probe sets (data not shown). Intriguingly, high *CIP2A* mRNA expression correlates significantly with reduced disease-free survival (DFS) (Fig. S1A, B). In separate analyses of patients in Union for International Cancer Control (UICC) stage I (tumour infiltration to muscularis propria), II (tumour infiltration beyond muscularis propria) or III (lymph node metastasis), only patients in stage UICC III showed a significant correlation between *CIP2A* expression and DFS. In comparison, patients in stage UICC I and II showed only a slight correlation between low *CIP2A* expression and prolonged survival that did not reach statistical significance. This may be due to the small number of samples and fewer relapse events in comparison to patients in stage UICC III (Fig. S2A–C). As DFS could not be reached in stage UICC IV (distant metastases), it was not possible to analyse the correlation of *CIP2A* expression to the survival of patients in stage UICC IV with this data set.

**Figure 1:**
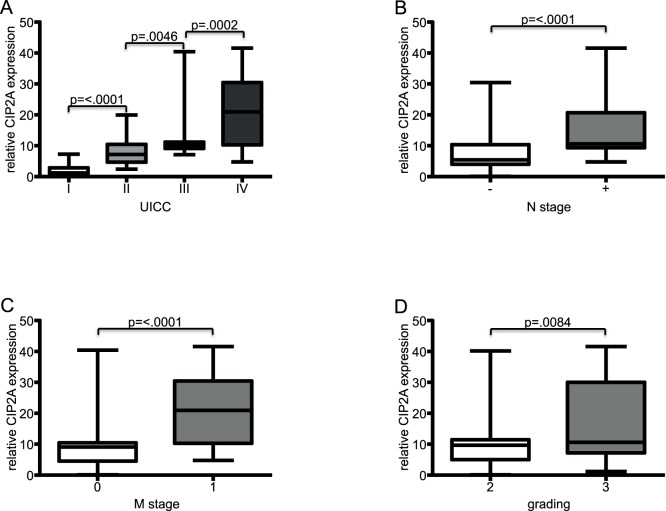
*CIP2A* mRNA expression is significantly correlated with advanced tumour stage. The panels show box and whisker plots documenting relative *CIP2A* mRNA levels in tumors stratified according to UICC stage (A) (UICC I vs. II p<.0001; II vs. III p = .0046; III vs. IV p = .0002), according to lymph node metastasis (B; N- vs. N+ p<.0001), according to distant metastasis (C; M0 vs. M1 p<.0001), and according to histological grading (D: G2 vs. G3 p = .0084).

We further analysed *CIP2A* mRNA levels in an independent set of 104 colorectal cancer tissue samples using an RT-QPCR approach. The expression of *CIP2A* mRNA was determined relative to two housekeeping genes, beta-2 microglobulin (*b2MG*) and glyceraldehyde-3-phosphate dehydrogenase (*GAPDH*). The expression levels were compared to the median *CIP2A* mRNA expression in normal mucosal tissue. *CIP2A* expression relative to *b2MG* or relative to *GAPDH* showed a high correlation (R^2^ = 0.86) (Fig. S3). Consistent with previous findings on CIP2A expression in cancer, we found *CIP2A* mRNA expression in 93 of 104 (89.4%) cancer samples to be at least four times higher than in normal colon mucosal tissues. Furthermore, *CIP2A* expression level was significantly correlated to the UICC stage, lymph node metastasis, distant metastasis, and histological tumour grading (Fig. 1). We found no association between *CIP2A* expression and patient age, gender or cancer location (right vs. left colon) (Table S2).

### CIP2A mRNA Expression and Postoperative Survival of Colon Cancer Patients

**Figure 2:**
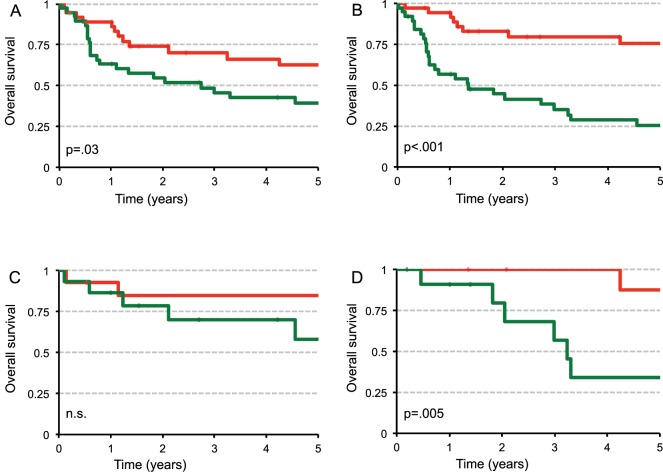
Patients with *CIP2A* high mRNA expression have an overall lower survival rate than patients with *CIP2A* low mRNA expression. The graphs show Kaplan–Meier curves of OS according to *CIP2A* mRNA expression. (red: *CIP2A* mRNA expression below median fold expression value of 10,5 above normal tissue), green: *CIP2A* mRNA expression above median fold expression value of 10,5 above normal tissue) (**A & B**) All patients with respect to *CIP2A* mRNA expression normalized to housekeeping gene (n = 75) (A: b2MG; B: GAPDH) (**C**) Patients in Stage UICC II with respect to *CIP2A* mRNA expression normalized to housekeeping gene GAPDH (n = 29) (**D**) Patients in Stage UICC III with respect to *CIP2A* mRNA expression normalized to housekeeping gene GAPDH (n = 21).

Overall survival (OS) was used for survival analysis. Overall survival was defined as the time interval between surgery and tumour-associated death. Univariate 1-, 3-, and 5-year OS analyses revealed that patients with an advanced primary tumour stage, lymph node metastasis, distant metastasis, advanced UICC-stage, and high histological grades had worst outcomes (Table 1). For further analysis, patients were divided into two groups, with *CIP2A* expression above (high) or below (low) median value. Patients with high *CIP2A* mRNA expression had significantly lower OS than those with low *CIP2A* mRNA expression (Fig. 2A, B). Comparing patients in UICC stages I-IV separately with high and low *CIP2A* mRNA expression, only in the UICC III stage, patients with high *CIP2A* levels had a reduced OS (Fig. 2C, D). In addition, a multivariate analysis indicated that an advanced UICC-stage and high *CIP2A* expression (*CIP2A* above median and *CIP2A* expression used as a continuous marker) were independent prognostic factors for poor outcome in patients with colon cancer (Table 2).

**Table 1:** Univariate 1, 3, 5-year overall survival of CRC patients.

		1-Year (n = 74)			3-Year (n = 58)			5-Year (n = 55)		
Parameters		Total	overall survival	p-value	Total	overall survival	p-value	Total	overall survival	p-value
*T stage*	T_1,2_	9	100%	<0.0001	5	80.0%	0.0014	4	75.0%	0.0004
	T_3,4_	64	75%		53	59.6%		50	52.0%	
*N Stage*	N_0_	32	93.8%	<0.0001	23	82.6%	<0.0003	22	77.3%	<0.0004
	N_1,2_	41	65.9%		34	47.1%		32	37.5%	
*M stage*	M_0_	50	100%	<0.0001	36	94.4%	<0.0001	33	84.9%	<0.0001
	M_1_	23	30.4%		21	4.8%		21	4.8%	
*UICC stage*	I-II	29	100%	<0.0001	20	95.0%	<0.0001	19	89.5%	<0.0001
	III-IV	44	63.6%		37	43.2%		35	34.3%	
*Histological grade*	G_2_	56	83.9%	<0.04	42	66.7%	<0.05	40	60.0%	0.0506
	G_3_	17	58.8%		15	46.7%		14	35.7%	
*CIP2A expression*	< median	32	100%	<0.0001	22	95.5%	<0.0001	20	95.0%	<0.0001
	> median	41	61.0%		34	41.2%		34	29.4%	

**Table 2:** Cox regression analysis in predicting the overall survival of CRC patients.

Risk factors	HR	95% CI	*p*-value
*UICC stage*	2.38	1.38–4.1	<0.01
*Histological grade*	1.03	0.49–2.16	n.s.
*CIP2A expression (above median vs. below median)*	3.06	1.27–7.37	<0.05
*CIP2A expression (continues factor)*	1.02	1.0–1.05	<0.02

(HR Hazard ratio).

**Figure 3:**
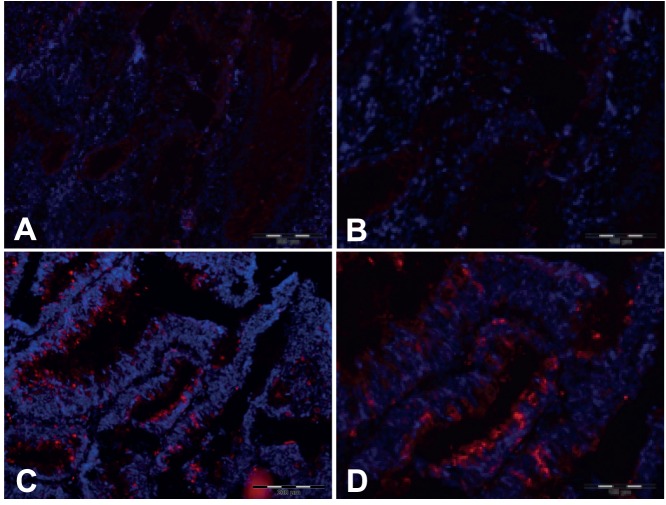
CIP2A protein levels in colon cancer correlate with *CIP2A* mRNA expression. The panels show representative examples of immunofluorescence staining, showing CIP2A protein expression in cancer cells of patients with low (A+B) or high *CIP2A* (C+D) mRNA expression (A+C x100, B+D x200 magnification).

To test whether *CIP2A* mRNA expression is correlated with CIP2A protein expression in CRC, ten normal mucosa tissue sections, four CRC tissue samples expressing low *CIP2A* mRNA levels and five tissue samples expressing high *CIP2A* mRNA levels were stained for CIP2A protein expression. In normal mucosa tissue sections, no or only a very weak staining for CIP2A could be detected (data not shown). Cancers expressing low levels of *CIP2A* mRNA showed a weak staining for CIP2A protein, whereas cancers expressing high *CIP2A* mRNA levels showed a strong staining for CIP2A protein. This results show that CIP2A protein and mRNA levels correlate closely *in vivo*. (Fig. 3).

### Effects of CIP2A Depletion on Cell Growth and Myc Expression in CRC

**Figure 4:**
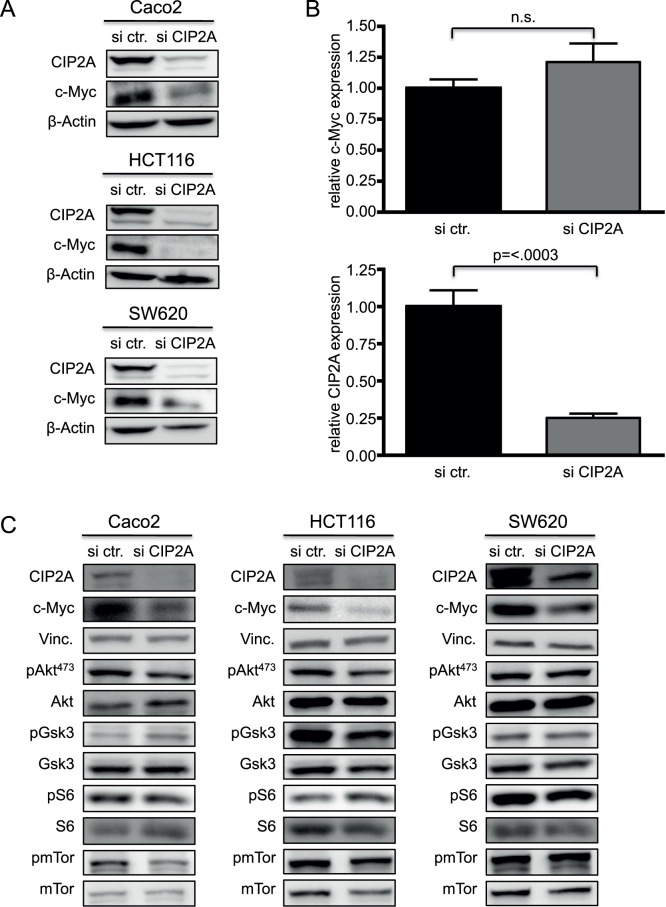
Depletion of CIP2A downregulates c-Myc protein expression in colon cancer cells. (**A**) Immunoblot analysis of CIP2A and c-Myc protein expression in Caco2, HCT116 and SW620 cells transfected with siRNA targeting CIP2A or control siRNA. Cells were harvested 72 h after transfection (n = 3 for each cell line). (**B**) Real-time PCR analysis of *CIP2A* and *c-Myc* mRNA expression in HCT116 cells transfected with siRNA targeting CIP2A or control siRNA (n = 3). (**C**) Depletion of CIP2A does not change activation status of AKT or its downstream targets. The panels show immunoblots of the indicated proteins and phosphorylated proteins (p) in Caco2, HCT116 and SW620 cells transfected with siRNA targeting CIP2A or control siRNA as before (n = 3 for each cell line).

We further wanted to clarify the influence of CIP2A on its target proteins in CRC cell lines. Protein expression of CIP2A was remarkably reduced in three colon cell lines (Caco2, HCT116 and SW620) after treatment with specific siRNA against *CIP2A*. Furthermore, CIP2A knockdown led to substantial reductions in c-Myc levels in all three cell lines (Fig. 4A; n = 3 for each cell-line). This was due to posttranscriptional regulation of c-Myc protein levels, since we detected no change in *MYC* mRNA expression (Fig. 4B; n = 3). Previous studies indicated that CIP2A knockdown led to a reduced activity of Akt, shown by reduced phosphorylation at serine 473 (pAkt^473^), and thereby, inhibited the PI3K/mTor pathway. However, we did not detect any significant changes in phosphorylated Akt after CIP2A knockdown in Caco2, HCT116 or SW620 cells. Furthermore, phosphorylation of the downstream Akt targets, including pmTor, pS6, and pGsk3, was not significantly altered after knockdown of CIP2A (Fig. 4C; n = 3 for each cell line). Overall, this shows that c-Myc protein is under the control of CIP2A in CRC, whereas the PI3K/mTor appears to be independent of CIP2A.

**Figure 5:**
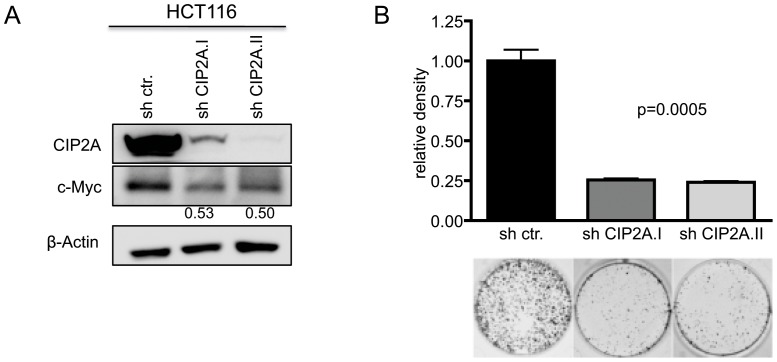
CIP2A is required for growth of HCT116 cells. (**A**) Immunoblot analysis of CIP2A and c-Myc protein expression in HCT116 cells infected lentiviral with shRNA targeting CIP2A or a ctr. shRNA. Numbers below lines indicate the c-myc protein expression relative to c-myc levels in control cells (n = 2). (**B**) Colony formation of HCT116 cells after 7 days. (*Top*), density of colonies stained with crystal violet; (*bottom*), representative of the indicated cultures (n = 3).

Because siRNAs are typically lost over time, and to exclude off-target effects, we tested two different shRNAs targeting *CIP2A* to evaluate the growth inhibiting effect of CIP2A knockdown. HCT116 cells were infected lentiviral with vectors carrying shRNAs against *CIP2A*. Immunoblot analyses showed substantial knockdown of CIP2A protein with corresponding reduction in c-Myc protein levels (Fig. 5A; n = 2). Accordingly, cell proliferation was markedly altered in cells infected with shRNA against *CIP2A* (Fig. 5B; n = 2).

### CIP2A Expression is Downstream of MEK Kinase in CRC

**Figure 6:**
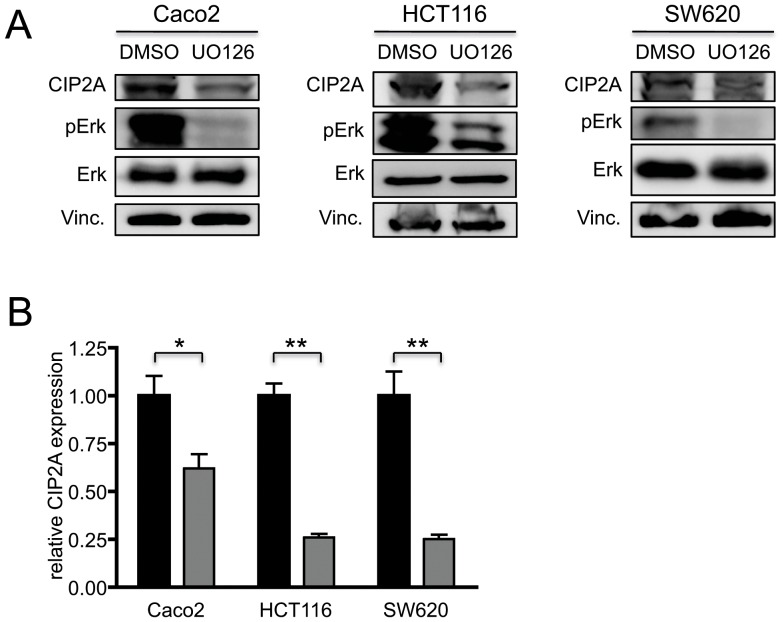
CIP2A expression is regulated by MAPK signalling. Caco2, HCT116 and SW620 cells were treated with DMSO or the MEK inhibitor UO126 for 24(n = 3 for each cell line). (**A**) Immunoblot analysis of CIP2A and p-ERK protein expression in Caco2, HCT116 and SW620. (**B**) Real-time PCR analysis of *CIP2A* mRNA expression (*<0.05; **<0.005).

To identify potential upstream regulators of *CIP2A* expression, we treated Caco2, HCT116 and SW620 cells with UO126, a well-characterised Mek1/2 inhibitor [16]. Treatment with UO126 for 24 h led to a significant reduction in *CIP2A* mRNA and CIP2A protein expression compared to DMSO treated cells (Fig. 6A, B; n = 3 for each cell line). This effect was more pronounced in cells that harboured a MAPK pathway mutation, such as SW620 (*KRAS*) and HCT116 (*KRAS*), than in Caco2 cells that are wild type for *KRAS*.

## Discussion

Recently “The Cancer Genome Atlas Network” demonstrated that deregulated c-Myc expression is a hallmark of virtually all CRCs, independent of the set of specific mutations that are present in each tumour [5]. For example, mutations in the tumour suppressor *APC* and the oncogene *KRAS* lead to an upregulation of *MYC* mRNA [5]. On posttranscriptional level, the loss of miR34 family by hypermethylation, which occurs in over 90% of all CRC, stabilizes *MYC* mRNA [17], [18]. Furthermore, a mouse model of colon cancer driven by loss of *APC* shows that colon tumour formation depends on continuous c-Myc expression [19].

So far, little is known about the posttranslational regulation of c-Myc levels in CRC. In the present study, we demonstrated that *CIP2A* expression is elevated in colon carcinoma samples, compared to its expression in normal, large intestine tissue, and that *CIP2A* expression is negatively correlated to both OS and clinical pathological parameters. Since there are no validated cut-off points for *CIP2A* expression levels to clarify high vs. low expression groups, we used the median mRNA expression level as the cut-off point. Despite a relatively low number of patients in our study, and therefore a relatively low number of cancer-related deaths in subgroup analyses, we could demonstrate a significant correlation of *CIP2A* levels with tumour-associated survival.

Two previous studies analysed expression levels of CIP2A in CRC and demonstrated enhanced levels in CRC relative to normal mucosa, consistent with the results reported here [13], [14]. Furthermore, a close correlation between CIP2A and Myc levels has been noted before [3], [13] and we show here that elevated levels of CIP2A are required to maintain Myc expression in CRC cell lines. Surprisingly, correlation with OS was seen in only one of the previous studies [14]. While we do not know the precise reason for the discrepancy, we note that both Teng et al. [14] and our study assigned a relatively high percentage of patients to the low expression group (68 and 50% respectively), whereas Böckelman et al. [13] defined only 15% of patients as low CIP2A expression tumours. We suggest that these differences in the stratification of patients according to CIP2A levels may account for the different results. The notion that CIP2A is overexpressed in colon cancer, thereby enhancing Myc protein levels and influencing tumour related survival, is consistent with previous results in other solid tumours [6]; [7]; [8]; [9]. A prospective study will be required to evaluate if CIP2A expression on mRNA or protein level can serve as a predictive marker for the survival of patients with CRC.

It was shown previously that CIP2A stabilises c-Myc by inhibiting its PP2A-mediated degradation in tumour cells [3]. Consistent with this model, the present study shows that downregulation of the c-Myc protein is observed when CIP2A is silenced with siRNAs or shRNAs in colorectal carcinoma cells. Due to the fact that we used cell lines harbouring different mutations for oncogenic pathway commonly mutated in CRC (Caco2: APC^mut^, KRAS^wt^, Pi3K^wt^; HCT116: APC^wt^, ß-catenin^mut^, KRAS^mut^, Pi3K^mut^; SW620: APC^mut^, KRAS^mut^, Pi3K^wt^), we postulate that the dependence of c-Myc protein expression on CIP2A is independent of the presence of mutations in WNT, PI3K/mTor or MAPK-pathways, but this will need to be evaluated further. We also found that shRNA-mediated depletion of CIP2A in HCT116 colon carcinoma cells markedly reduced their growth potential. This is consistent with reports that CIP2A inhibition resulted in growth arrest and diminished clonogenic potential in several other malignancies [6]; [7]; [8].

PP2A dephosphorylates pAkt and CIP2A enhances PI3K/mTor signalling by inhibiting PP2A mediated dephosphorylation of pAKT in hepatocellular carcinomas (HCC) [20]; [21], [22]. Our results show that depletion of CIP2A expression in colorectal cancer cells does not affect Akt signalling, in contrast to observations in HCC cells. We also found no or only minor changes in the pAkt levels or known Akt downstream targets. Most likely, therefore, PP2A activity towards some of its substrates (Akt) differs between different tissues.

Little is known about the mechanism underlying the enhanced expression of CIP2A in malignant cells. In gastric cancer, *helicobacter pylori* infections up-regulate *CIP2A* expression through a Src/Erk dependent pathway [11]. The human papillomavirus 16E7 oncoprotein upregulates *CIP2A* in cervical cancer [12]. It is currently unclear what contributes to CIP2A overexpression in colon cancer. Two findings support the hypothesis that CIP2A expression in colon cancer is downstream of the MAPK pathway. First, previous work showed that CIP2A expression is positively correlated with EGFR expression [13]. The MAPK pathway is one of the major targets of EGF signalling. Second, we showed that CIP2A is downregulated after inhibiting the MAPK pathway in three colon carcinoma cell lines; downregulation in cell lines harbouring a *KRAS* mutation (HCT116, SW620) is more pronounced than in Caco2 not harbouring a MAPK-pathway mutation. Induction of CIP2A may, therefore, be a critical mediator that links deregulated mitogenic signalling to enhanced c-Myc expression in CRC.

In conclusion, the results of this study show that *CIP2A* is associated with colorectal cancer related survival. High expression of *CIP2A* mRNA is correlated with reduced DFS and OS. Therefore, *CIP2A* represents a new prognostic factor in the diagnosis of colorectal cancer. In addition, CIP2A may be a promising therapeutic target in the development of therapies for colorectal cancer.

## Supporting Information

Figure S1 **Kaplan–Meier curves of disease free survival of all patients (n = 226) with respect to** ***CIP2A*** **mRNA expression status in published microarray analysis.** (A & B) DFS of all patients according to the microarray probe set.
(EPS)

Figure S2 **Disease free survival of patients with colon cancer in UICC I-III stage, grouped according to** ***CIP2A*** **mRNA expression status.** (**A**) UICC I; (**B**) UICC II; (**C**) UICC III.
(EPS)

Figure S3 **Linear regression analysis of relative** ***CIP2A*** **mRNA expression normalized to two housekeeping genes, b2MG and GAPDH (n = 104; R^2^ = 0.86).** The linear regression is highly significant (p<0.001).
(PSD)

Table S1 Primer sequences.
(DOCX)

Table S2 Characteristics of patients with CRC and association between *CIP2A* expression and clinicopathologic variables (lymphovascular invasion and location was documented for 100 patients).
(DOCX)
