# Supplementary material for: CIP2A Influences Survival in Colon Cancer and Is Critical for Maintaining Myc Expression
Source: PLoS One. 2013 Oct 1;8(10):e75292. doi: 10.1371/journal.pone.0075292 (PMC3788051; doi:10.1371/journal.pone.0075292)
Supplement: Table S1 — Primer sequences. (DOCX) [file pone.0075292.s004.docx]

Supplementary Table 1: Primer sequences

| Gene Name | Primer sequnece |
| --- | --- |
| shCIP2A I | (5´CCGGGCTAGTATGTTGAGAGAAGTTCTCGAGAACTTCTCTCAACA3’) |
| shCIP2A II | (5´CCGGGCTAGTAGACAGAGAACATAACTCGAGTTATGTTCTCTGTC3’) |
| CIP2A | For GAACAGATAAGAAAAGAGTTGAGCATT  Rev CGACCTCTAATTGTGCCTTTT |
| MYC | For CACCAGCAGCGACTCTGA  Rev GATCCAGACTCTGACCTTTTGC |
| B2MG | For GTGCTCGCGCTACTCTCT  Rev GTCAACTTCAATGTCGGAT |
| GAPDH | For GCTCTGCAGGAGACAAGACC  Rev AGCATAGCTCACGCCCTCT |
