# Supplementary material for: CIP2A Influences Survival in Colon Cancer and Is Critical for Maintaining Myc Expression
Source: PLoS One. 2013 Oct 1;8(10):e75292. doi: 10.1371/journal.pone.0075292 (PMC3788051; doi:10.1371/journal.pone.0075292)
Supplement: Table S2 — Characteristics of patients with CRC and association between CIP2A expression and clinicopathologic variables (lymphovascular invasion and location was documented for 100 patients). (DOCX) [file pone.0075292.s005.docx]

Supplementary Table 2: Characteristics of patients with CRC and association between CIP2A expression and clinicopathologic variables (Lymphovascular invasion and location was documented for 100 patients)

| **Variable** | **Total**  **(n=104)** | **Relative CIP2A**  **expression** | | ***P*-value** |
| --- | --- | --- | --- | --- |
| *Gender*  Male  Female | 57  47 | 16.2 +/- 15.0  22.1 +/- 20.2 | n.s. | |
| *Age, years (median 70.5)*  < 70,5 á  > 70,5 á | 52  52 | 20.8 +/- 18.6  16.9 +/- 16.7 | n.s. | |
| *T stage*  T_1,2_  T_3,4_ | 19  85 | 6.7 +/- 4.7  21.6 +/- 18.4 | < 0.0001 | |
| *N Stage*  N_0_  N_1,2_ | 53  51 | 11.7 +/- 13.3  26.3 +/- 18.8 | < 0.0001 | |
| *M stage*  M_0_  M_1_ | 77  27 | 12.4 +/- 11.7  37.3 +/- 19.1 | < 0.0001 | |
| *Lymphovascular invasion*  No  Yes | 68  32 | 13.8 +/- 13.6  27.2 +/- 20.6 | 0.0017 | |
| *UICC stage*  I-II  III-IV  *Histological grade*  G_2_  G_3_ | 49  55  83  21 | 9.2 +/- 8.9  27.5 +/- 19.2  16.4 +/- 16.0  28.5 +/- 21.2 | < 0.0001  0.022 | |
| *Location*  Right colon  Left colon | 54  46 | 13.9 +/-12.4  21.3 +/- 19.2 | n.s. | |
